# Enhancing the Thermostability of *Bacillus licheniformis* Alkaline Protease 2709 by Computation-Based Rational Design

**DOI:** 10.3390/molecules30051160

**Published:** 2025-03-04

**Authors:** Yuan Yuan, Guowei Zhao, Jing Lu, Lei Wang, Yawei Shi, Jian Zhang

**Affiliations:** 1 College of Chemistry and Chemical Engineering, Shanxi University, Taiyuan 030006, China; 202112910002@email.sxu.edu.cn (Y.Y.); 202122911025@email.sxu.edu.cn (G.Z.); 2 College of Life Sciences, Shanxi University, Taiyuan 030006, China; jinglu@sxu.edu.cn; 3 Key Laboratory of Chemical Biology and Molecular Engineering, Ministry of Education, Institute of Biotechnology, Shanxi University, Taiyuan 030006, China; wanglei1007@sxu.edu.cn; 4 Shanxi Province Detergent Alkaline Protease Industrialization Key Technology and Application Engineering Research Center, Taiyuan 030006, China

**Keywords:** alkaline protease, rational design, thermal stability, deamidation reaction

## Abstract

The alkaline protease from *Bacillus licheniformis* strain 2709 (AprE 2709) is widely used in Chinese industries but faces stability challenges under high-temperature conditions. This study employed molecular modeling and mutagenesis to identify Asn residues at positions 61, 160, and 211 as key sites affecting the stability of AprE 2709. By leveraging the additive and cooperative effects of mutations, the mutant enzyme AprE 2709 (N61G/N160G/N211G) was engineered, exhibiting enhanced thermostability and catalytic activity. The mutant demonstrated a 2.89-fold increase in half-life at 60 °C and a 1.56-fold improvement in catalytic efficiency compared to the wild-type enzyme. Structural analysis revealed that the improved thermostability was due to altered electrostatic interactions and strengthened hydrophobic contacts. Targeting Asn residues prone to deamidation presents a promising strategy for improving protein heat tolerance. These findings not only enhance our understanding of enzyme stability but also lay a foundation for future research aimed at optimizing alkaline proteases for diverse industrial applications, particularly in high-temperature processes.

## 1. Introduction

In the food industry, proteases play a crucial role in various industrial processes [1,2]. In cheese-making, proteases facilitate curd formation and contribute to flavor development. In protein-rich beverages such as soy milk, proteases hydrolyze proteins into smaller peptides and amino acids, enhancing digestibility and bioavailability [3,4,5]. Additionally, in bakery products, proteases modify dough properties, leading to improved texture, softness, and volume. Proteases are categorized into acidic, neutral, and alkaline types. In China, the alkaline protease enzyme from *Bacillus licheniformis* strain 2709 (AprE 2709) is the most commonly used industrial protease [6,7,8,9,10]. While current research primarily focuses on the protein expression of AprE 2709, less attention has been given to its structural and functional properties [9,10]. Despite its widespread use, AprE 2709 exhibits limited thermostability. This restricts its application in the food industry and other sectors.

Advancements in structural and computational biology have made protein structure determination more efficient, utilizing computational prediction and experimental methods. Many successful rational designs for protein stability focus on engineering interior residues. For instance, the serine protease SP’s three-dimensional structure was predicted using the I-TASSER server, and the predicted model was subsequently refined using the online ModRef server to minimize side chain clashes. The refined model was then analyzed using the FireProt server, where stabilizing mutations were predicted based on evolutionary and energy-based approaches. The A29G and V366I mutations enhanced the K_cat_/K_m_ ratio by 1.5- and 1.8-fold, respectively, compared to the wild type [11]. Molecular dynamics simulations serve as a valuable tool for identifying optimal mutation sites. For example, mutations D28N and D116N in the metalloprotease PT121 from *Pseudomonas aeruginosa* extended half-lives at 60 °C by 1.07- and 1.8-fold, respectively, compared to the wild type (WT) [12]. Similarly, the keratinase activity of the KerZ1 T77C/A128D mutant at 60 °C increased by 46% compared to KerZ1 WT [13]. Using the PoPMuSiC algorithm, the predicted changes in the free energy of amino acid substitutions (ΔG) guided the design of keratinase tetra mutants (N122Y, N217S, A193P, and N160C) from *Bacillus licheniformis* BBE11-1, resulting in an 8.6-fold increase in half-life [14].

Deamidation is a hydrolytic reaction that removes the amide group from asparagine residues, converting them into aspartate. This modification alters the side-chain properties of the residues, which can disrupt protein structure and function, potentially affecting the protein’s stability and its secondary and tertiary structures. Deamidation-induced conversion of asparagine to aspartate can trigger local structural changes, potentially impacting overall protein stability. The N37D mutation in γS-crystallin reduced thermal stability and altered the formation of specific intermolecular hydrogen bonds [15]. Differential scanning calorimetry experiments showed that the N26D/N131D/N139D SOD1 mutant exhibited lower thermal stability than WT SOD1, confirming that deamidation of asparagine to aspartate destabilizes copper–zinc superoxide dismutase (SOD1) [16]. Deamidation introduces negative charges on the residues, thereby altering protein–protein interactions, which may affect activity or refolding [17]. Elevated temperatures can induce chemical modifications in proteins. Deamidation of asparagine and glutamine is a prominent non-enzymatic reaction that is significantly accelerated at elevated temperatures and physiological pH [18]. Non-enzymatic deamidation of Asn residues has been reported in over 200 types of naturally occurring peptides and proteins. Asn exhibits approximately 10-fold higher susceptibility to deamidation than Gln [19]. Enzyme stabilization can be enhanced by substituting Asn/Gln residues prone to deamidation, particularly those that significantly impact structural integrity [20]. Previous studies have demonstrated that Asn mutations can significantly improve enzyme thermal stability. For instance, the mutC triple mutant (N51E, N82H, N174S) of *B. subtilis* lipase 6B retained nearly twice the residual activity of the wild-type enzyme after four cycles at 90 °C [21]. However, the rational design of protease mutations based on deamidation remains largely unexplored. This research gap underscores the need for targeted strategies to improve the stability of proteases such as AprE 2709.

AprE 2709 contains 19 asparagine (Asn) residues in its structure. In this study, a computer-aided rational design strategy was employed to introduce Asn mutations and enhance the stability of AprE 2709. Molecular dynamics (MD) simulations were performed to identify highly flexible Asn residues in the AprE 2709 structure. Three specific sites (Asn61, Asn160, and Asn211) were identified as key targets for modification. The mutant proteases were heterologously expressed in *B. subtilis* WB600. The hydrolytic stability and structural properties of the expressed proteases were subsequently assessed. Further MD simulations were performed to elucidate the molecular mechanisms underlying the enhanced stability of these mutants. This comprehensive approach provided valuable insights into the structural behavior of the modified proteases under hydrolytic conditions. This study investigated the potential of replacing deamidation-prone Asn residues to enhance the thermostability of AprE 2709.

## 2. Results

### 2.1. Three-Dimensional Structure Prediction of AprE 2709 (WT) Using AlphaFold 3 and SWISS-MODEL

Due to the absence of a crystal structure for AprE 2709 (WT), homology modeling was performed using AlphaFold3 (https://alphafoldserver.com/) and SWISS-MODEL (https://swissmodel.expasy.org/) to guide its rational design. A BLAST (https://blast.ncbi.nlm.nih.gov/Blast.cgi, accessed on 2 March 2025) search revealed that the crystal structure of subtilisin Carlsberg (PDB ID: 1C3L) exhibited the highest similarity to AprE 2709, with a sequence identity of 99%. Thus, the crystal structure of subtilisin Carlsberg was used as a template in SWISS-MODEL. The secondary and tertiary structures predicted by AlphaFold3 and SWISS-MODEL are presented in Figure S1. Both predictive approaches indicated that AprE 2709 (WT) consists of 9 α-helices and 16 β-sheets. The predicted structures from AlphaFold3 and SWISS-MODEL exhibited a high degree of consistency.

The reliability of the model was assessed using multiple validation tools, including PROCHECK, Verify-3D, and PROSA. The Ramachandran plot, a standard tool for homology model evaluation, indicated that 90.8% of residues were in favored regions, 8.8% in additionally allowed regions, and 0.4% in generously allowed regions (Figure S2). Verify-3D analysis (Figure S3) revealed that 98.56% of amino acid residues achieved an average 3D/1D compatibility score of ≥0.2. PROSA analysis of the AprE 2709 (WT) model yielded a Z-score of −10.37 (black dot), which falls within the acceptable range for structures determined by X-ray crystallography and NMR spectroscopy (Figure S4). The three evaluation methods provided consistent results in analyzing the three-dimensional structure of AprE 2709 (WT).

### 2.2. Identification of Potential Mutation Sites in AprE 2709 (WT) Through Amino Acid Fluctuation and Spatial Position Analysis

**Figure 1:**
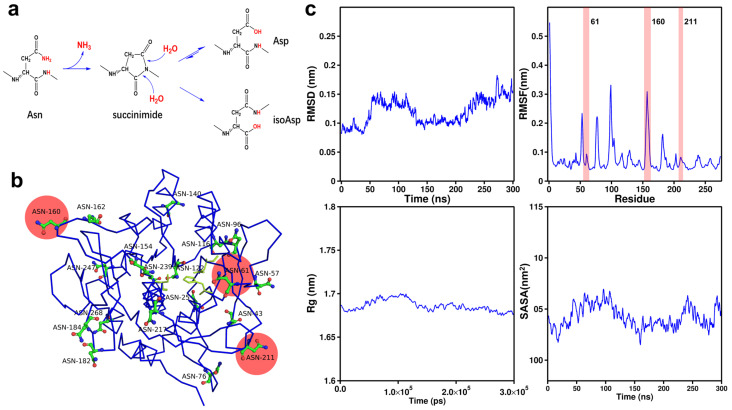
The location of the mutation targets. (**a**) The schematic diagram of deamidation reaction of AprE 2709 (WT) in aqueous solution; (**b**) The positional distribution of the 19 asparagines in AprE 2709 (WT) structure; (**c**) root-mean-square deviation (RMSD), root-mean-square fluctuation (RMSF), radius of gyration (Rg) and Solvent-Accessible Surface Area (SASA) values versus residues of AprE 2709 (WT) at 298.15 K.

Asn residues are prone to deamidation at elevated temperatures (Figure 1a), which consequently shortens the half-life of AprE 2709 (WT) [22]. Deamidation of asparagine (Asn) is a chemical process in which the amide group on its side chain is hydrolyzed under specific conditions, converting Asn into aspartic acid (Asp). This reaction can have a substantial impact on protein structure and stability.

To reduce the susceptibility to deamidation and improve the stability of AprE 2709 (WT), we explored substituting Asn with alternative amino acids. The relatively bulky side chain of Asn can induce steric hindrance, which may be alleviated by substituting it with smaller amino acids, potentially enhancing protease stability [23]. Glycine, known for its minimal molecular size and simple structure, emerged as an optimal candidate for substitution [24]. Serine, with its small backbone and polar hydroxyl group, facilitates hydrogen bonding [25]. This hydroxyl group not only enhances its solubility in aqueous environments by interacting with water molecules, but also enables interactions with other polar molecules or ions. Consequently, in this study, Asn residues were mutated to glycine and serine to investigate their impact on the stability of AprE 2709 (WT).

Identifying Asn residues suitable for mutation is essential for targeting specific modifications. We aimed to mutate key Asn residues in engineered AprE 2709 (WT) to investigate their effects on activity and stability. The complete structure of AprE 2709 (WT) was visualized using PyMOL, revealing 19 Asn residues, including Asn25, Asn43, Asn57, Asn61, Asn76, Asn96, Asn116, Asn122, Asn140, Asn154, Asn160, Asn162, Asn182, Asn184, Asn211, Asn217, Asn247, Asn239, and Asn268 (Figure 1b). To investigate this instability, molecular dynamics simulations (RMSD, RMSF, Rg, and SASA analyses) were performed on AprE 2709 (WT) (Figure 1c). The RMSF values for each residue, determined by tracking the fluctuations of individual amino acids in aqueous solution, indicated that higher RMSF values correspond to increased fluctuations at specific sites (Table 1) [26].

**Figure 2:**
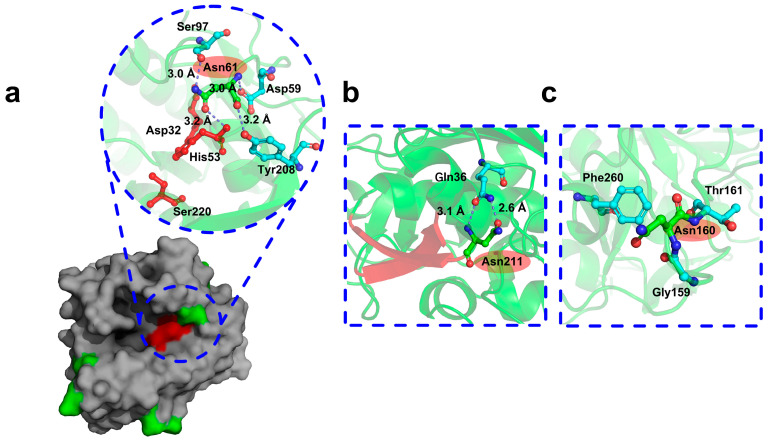
Identification of AprE 2709 mutation regions and sites. Green stick presentation represents the selected ASN61, ASN211, and ASN160. (**a**) The asparagine around the catalytic structural domain had only Asn61 sites, The red surface area indicates the catalytic active center; (**b**) Asn211 located between two β-folds; (**c**) no hydrogen bonding interactions with surrounding amino acids near site Asn160.

In AprE 2709 (WT), the catalytic triad (Figure 2) is composed of Asp32, His63, and Ser220. Among the 19 Asn residues, only Asn61 was near the catalytic domain (Figure 2). The catalytic domain is a crucial region indispensable for the protein’s function, with surrounding amino acids playing a pivotal role in preserving its correct conformation. Mutating Asn residues near the catalytic domain can enhance interactions with surrounding amino acids, stabilizing the three-dimensional structure and ensuring that the protein maintains its active conformation under varying conditions. Recent studies have shown that appropriate mutations within or near the active site and substrate binding regions can improve both the thermostability and activity of industrial enzymes [27,28,29]. As a result, Asn61 was selected as the first mutation site due to its proximity to the catalytic domain and its high fluctuation. Among all the Asn residues in AprE 2709, only those at positions 211 and 239 are located between two secondary structural elements. The Asn residue at position 239 exhibited an RMSF value of 0.0898 nm, while the Asn residue at position 211 exhibited an RMSF value of 0.0832 nm (Table 1). However, Asn239 is located between two α-helices, while position 239 is located between two α-helices. β-sheet regions are generally more flexible and dynamic than α-helical regions. Mutating residues in β-sheet regions may have a more significant effect on protein stability and function due to their inherent structural flexibility. In contrast, α-helices are generally more stable and rigid, and mutations in these regions may disrupt the α-helix structure, potentially leading to more complex or unpredictable effects on overall protein stability. To avoid excessive destabilization of the protein structure, position 211 was chosen as the more suitable target for mutagenesis. Thus, Asn211, located between two β-sheets, was chosen as the second mutation site (Figure 2). The Asn residues exhibiting the highest fluctuations in AprE 2709 (WT) are situated at positions 76 and 160. While the residue at position 76 forms hydrogen bonds with surrounding amino acids, stabilizing its position, the residue at position 160, located in the surface loop region of the protease, does not form such interactions. The lack of stabilizing hydrogen bonds in this flexible surface region, coupled with its high fluctuation in molecular dynamics simulations, makes Asn160 a key candidate for mutation to investigate its impact on protease stability (Figure 2). Ultimately, Asn61, Asn160, and Asn211 were identified as the critical Asn residues for targeted mutation.

### 2.3. Biochemical Characterization of AprE 2709 Mutants

Focusing on Asn 61, Asn 160, and Asn 211, we subjected these positions to single-point mutations, including AprE 2709 (N61S), AprE 2709 (N160S), AprE 2709 (N211S), AprE 2709 (N61G), AprE 2709 (N160G), and AprE 2709 (N211G). Epistatic effects in proteins refer to the non-additive influence of amino acid mutations on protein folding and functionality. An epistatic interaction occurs when the combined impact of two mutations deviates from the sum of their individual effects. Numerous studies have demonstrated that strategically combining mutation sites influencing the same catalytic function can significantly enhance the catalytic efficiency of the target protein [30]. To enhance the catalytic performance of AprE 2709 (WT), we constructed a series of double and triple mutants, including AprE 2709 (N61S/N160S), AprE 2709 (N160S/N211S), AprE 2709 (N61S/N211S), AprE 2709 (N61S/N160S/N211S), AprE 2709 (N61G/N160G), AprE 2709 (N160G/N211G), AprE 2709 (N61G/N211G), and AprE 2709 (N61G/N160G/N211G). As a result, we constructed 14 mutants (M1–M14; Table 2).

Alkaline proteases, widely used in the food industry, typically function between 40 °C and 60 °C. For example, in tofu production, alkaline protease improves both texture and flavor, often at around 60 °C. In meat tenderization, it is used at similar temperatures to enhance tenderness, while in dairy processing, such as cheese production, it facilitates protein breakdown and maturation at 60 °C. Therefore, AprE 2709 (WT) and the 14 mutants were incubated at 60 °C to assess their respective half-lives (T_1/2_). Residual enzyme activity was used as the screening parameter to identify proteases with improved thermal stability (Table 2). The half-life of AprE 2709 (WT) at 60 °C was 19 min. Among the mutants, M14 exhibited significantly improved thermostability, with a half-life of 74 min at 60 °C—an enhancement of 2.89-fold over AprE 2709 (WT). Mutant M7 also showed a prolonged half-life of 62 min. The half-life of M3 increased by 1.84-fold compared to AprE 2709 (WT) after the single mutation of Asn to Ser at position 211.

**Figure 3:**
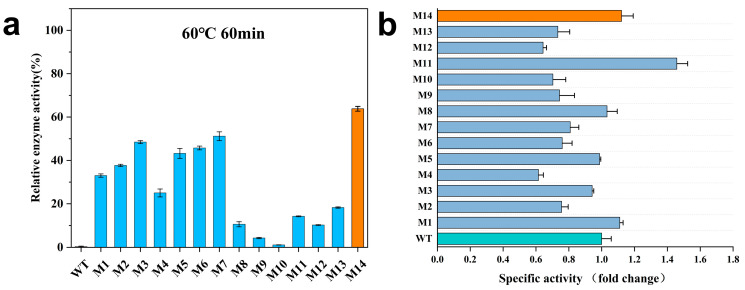
(**a**) Residual enzyme activity after incubation of AprE 2709 (WT) and mutants at 60 °C for 60 min; (**b**) determination of enzyme specific activity in AprE 2709 (WT) and mutants.

The residual enzyme activity of AprE 2709 (WT) and the 14 mutants was measured after incubation at 60 °C for 60 min (Figure 3a). After 60 min, AprE 2709 (WT) retained only 0.39% of its activity, whereas M14 exhibited superior stability, maintaining 63.81% of its activity. Mutant M7 retained 51.17% of its activity after 1 h at 60 °C. The specific activity of AprE 2709 (WT) and the mutants was measured using casein as the substrate at 40 °C (Figure 3b). Mutants M1, M8, M11, and M14 exhibited higher activity compared to AprE 2709 (WT). In protein modification, the simultaneous enhancement of thermostability and catalytic activity is often hindered by an activity–stability trade-off. In this study, the specific enzyme activity of M14 increased by 1.12-fold compared to AprE 2709 (WT), indicating that the modification of flexible sites within the protein can effectively mitigate the activity–stability trade-off during protein modifications. Further investigation of the mutants’ properties through kinetic experiments (Table 3, Figures S5 and S6) revealed that the Michaelis constant (K_m_) for M14 was lower than that for AprE 2709 (WT), indicating enhanced substrate affinity. The catalytic efficiency (K_cat_/K_m_) of the AprE 2709 M14 was higher than 1.56 times that of AprE 2709 (WT). Additionally, M14 had an optimal temperature 10 °C higher than that of AprE 2709 (WT). The pH optimum of M14 shifted from 10 to 11 toward more alkaline conditions.

### 2.4. Molecular Dynamics Simulation and Structural Analysis of the Triple Mutant AprE 2709 (N61G/N160G/N211G) for Enhanced Stability

**Figure 4:**
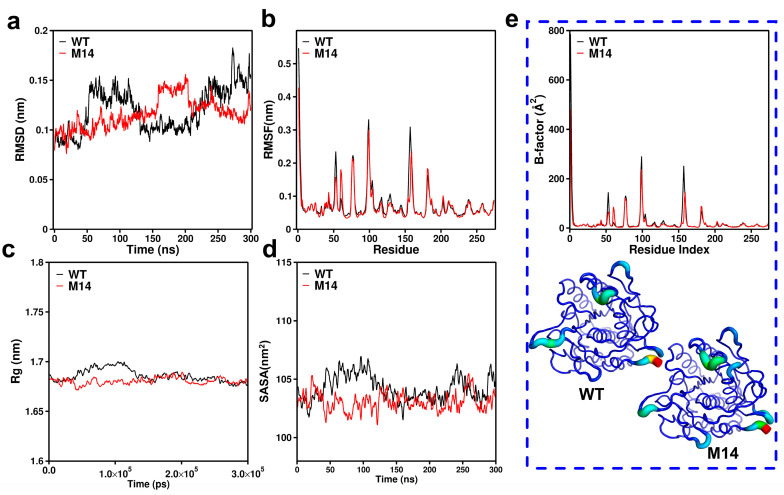
Molecular dynamics simulations for AprE 2709 (WT) and M14 of AprE at 298.15 K. (**a**) Root-mean-square deviation (RMSD) of WT and M14; (**b**) RMSF value of each residue in AprE 2709 (WT) and M14; (**c**) comparison of radius of gyration (Rg) between AprE 2709 (WT) and M14; (**d**) Solvent-Accessible Surface Area (SASA) of AprE 2709 (WT) and M14; (**e**) B-factor of AprE 2709 (WT) and M14. The highest B-factor position in each structure is colored red, and the lowest B-factor position is colored dark blue. The thickness of the protein backbone also is proportional to the B factors of Cα atoms.

The results above showed that the M14 mutant (AprE 2709 (N61G/N160G/N211G)) exhibits remarkable performance. To investigate the mechanism underlying the change in thermostability of the M14 mutant, we performed molecular dynamics simulations of both AprE 2709 (WT) and M14 at 298.15 K for 300 ns. The RMSD quantifies the cumulative atomic deviations between the conformation at a specific time point and the reference conformation. It is an important measure of system stability. After 300 ns of MD simulation, the average RMSD of M14 at 298.15 K was lower than that of AprE 2709 (WT). This indicated that M14 enhances the overall rigidity of the protease (Figure 4a). To further investigate changes in structural flexibility, we analyzed RMSF values for each amino acid. A lower RMSF value indicates a less flexible and more thermally stable region, suggesting that M14 has a more rigid and stable protein structure compared to AprE 2709 (WT) (Figure 4b). Additionally, the estimated Rg values of AprE 2709 at 298.15 K were compared between WT and M14. The results showed that mutant M14 exhibits a more compact structure and superior heat resistance compared to AprE 2709 (WT), as evidenced by a lower Rg value for M14 (Figure 4c). This confirmed that the overall protein structure of the mutant became more compact and its resistance to unfolding was enhanced following molecular modification. By monitoring the SASA changes of AprE 2709 (WT) and M14 over the 300 ns timescale, we gained a deeper understanding of their dynamic stability. As shown in Figure 4d, M14 displayed greater compactness and stability than AprE 2709 (WT), with consistently lower SASA values throughout the 300 ns simulation. Figure 4e further illustrated that mutation at the N61G, N160G, and N211G sites resulted in a significant decrease in the B-factor for M14, reflecting reduced atomic fluctuations and increased structural rigidity.

### 2.5. Mechanisms Underlying the Stability of AprE 2709 (N61G/N160G/N211G) Revealed by Molecular Dynamics Simulations

**Figure 5:**
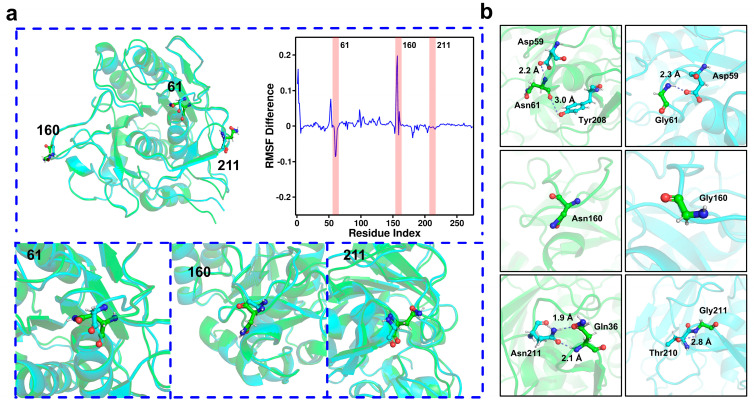
(**a**) Structural overlap between AprE 2709 (WT) and the M14 mutant. Green represents AprE 2709 (WT), and sky blue represents the M14 mutant. Enlarged images represent the structural backbone changes in the whole space before and after mutation of the three mutation sites (N61G, N160G, N211G). (**b**) Hydrogen bonding changes before and after mutations at sites 61, 160, and 211.

To investigate the mechanisms underlying the enhanced stability of the M14 mutant, we analyzed its structural modifications. By superimposing the models of AprE 2709 (WT) and the M14 mutant, we observed reduced steric hindrance following the mutation of Asn to glycine at positions 61, 160, and 211 (Figure 5a). Additionally, we calculated the difference in root-mean-square fluctuation (RMSF) values by subtracting the RMSF of the M14 mutant from that of AprE 2709 (WT) (Figure 5a), highlighting changes in flexibility at the mutation sites. The substitution of hydrophobic residues with large side chains by those with smaller side chains minimized steric hindrance in the catalytic pocket. This reduced surface tension and promoted favorable hydrophobic interactions among nonpolar residues, thereby enhancing both activity and thermal stability [31]. Five mutations were introduced in polyphosphate glucokinase: F30L, R34P, T72A, V88M, and A231T. This resulted in a significant increase in half-life and a 2.95-fold enhancement of activity. The substitution of bulky threonine with smaller hydrophobic alanine created additional space in the binding cavity for product release [32]. We also analyzed the hydrogen bonding patterns at positions 61, 160, and 211, both before and after the mutations (Figure 5b). Specifically, the mutation of Asn to glycine at position 61 resulted in a reduction in the number of hydrogen bonds. The number of hydrogen bonds at positions 160 and 211 remained unchanged before and after the mutations. Therefore, hydrogen bonding interactions were not the main reason for the increased stability of the M14 mutant.

**Figure 6:**
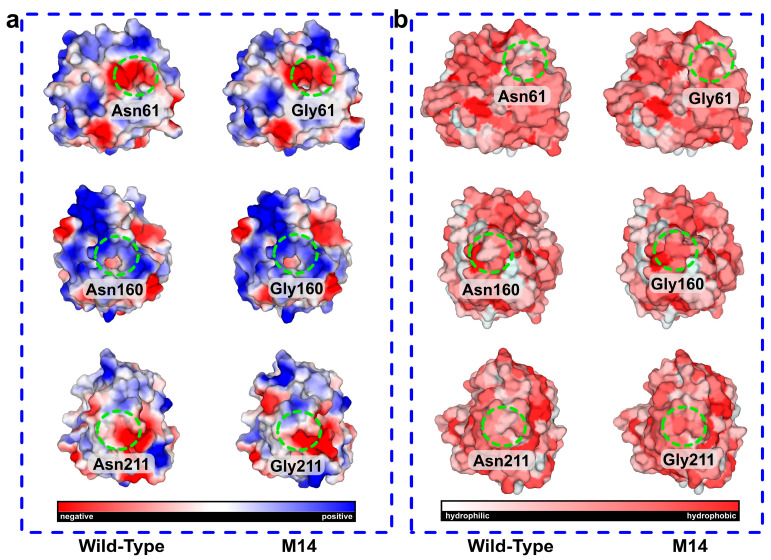
(**a**) Surface electrostatic potential changes in AprE 2709 (WT) and M14 mutants; (**b**) AprE 2709 (WT) and M14 analysis based on hydrophobic interactions. The redder colors in the graph represent more hydrophobicity.

Electrostatic potential energy calculations revealed that the N-to-G mutations induced a shift toward neutral charges at the mutation sites, promoting a more stable conformation (Figure 6a). These alterations in electrostatic potential enhanced the mutant’s ability to maintain a stable structure, thereby improving its thermal stability. The pathways of protein folding are significantly influenced by hydrophobic interactions formed between hydrophobic residues [33]. The introduction of multiple mutations in hydrophobic residues significantly enhances thermal stability, leading to effects that are substantially better than those of single mutations [34,35,36]. In the M14 mutant, the asparagine residues at positions 61, 160, and 211 were simultaneously mutated to the more hydrophobic glycine, providing an additional factor for thermal stability [37]. Hydrophobic residues are buried within the protein core, forming a hydrophobic core, and improving the packing of this core enhances the thermal stability of the protein [38,39]. Furthermore, the optimization of side chains of hydrophobic amino acids in the binding pocket contributes to improved substrate binding. In M14, the glycine at position 61 within the binding pocket plays a crucial role in enzyme catalytic efficiency. This also explained why the M14 mutant has no reduction in viability along with increased stability. When introducing hydrophobic residues near the active site, leucine exhibits good compatibility, which can enhance thermal stability by strengthening core packing and hydrophobic interaction clusters, as well as improving affinity and activity by increasing hydrophobic interactions with the substrate [40]. Figure 6b showed a significant increase in local hydrophobicity following the N-to-G mutations at the three sites. The clustering of hydrophobic residues inside the protein prevents unfavorable interactions with water, further stabilizing the protein’s stable conformation and increasing thermal stability. Cui et al. reported that the Q360C mutant, located in the central (b/a)8 barrel, significantly enhanced the hydrophobic interaction near position 360 to increase thermostability [41]. Overall, this reduction in hydrophilicity promotes the aggregation of hydrophobic amino acid residues, forming a tighter hydrophobic core. This interaction lowers the overall energy state of the protein, thereby enhancing its thermal stability.

## 3. Materials and Methods

### 3.1. Chemicals and Materials

The Mut Express II Fast Mutagenesis Kit V2 (C215, Vazyme, Nanjing, China) was used for site-directed mutagenesis. Folin–Ciocalteu phenol reagent was obtained from Sangon (Shanghai, China). Casein and trichloroacetic acid (TCA) were sourced from Aladdin Reagent Co., Ltd. (Shanghai, China). Gel purification, plasmid extraction, and recombination kits, along with other biochemical reagents, were sourced from Vazyme (Nanjing, China). Primers were synthesized by GenScript (Nanjing, China). Unless otherwise specified, all other chemicals and reagents were of analytical grade and commercially available.

### 3.2. Plasmid and Strains

The plasmid pBE2R was used for gene expression analysis. *Escherichia coli* DH5α strain was used as the host strain for cloning. The *B. subtilis* WB600 expression system was selected for protease expression due to its simplicity, rapid growth, and cost-effectiveness. *B. licheniformis* 2709 was preserved in our laboratory.

### 3.3. Protein Structure Prediction and Evaluation of AprE 2709

The complete amino acid sequence of AprE 2709 (GenBank: ABU68339.1) was retrieved from the National Center for Biotechnology Information protein sequence database. The protein structure was modeled using AlphaFold 3 and SWISS-MODEL [42,43] with subtilisin Carlsberg (PDB ID: 1c3l) serving as the template. The accuracy of the resulting model was confirmed by PROCHECK (Version 3.5.4) through a Ramachandran plot. The final AprE 2709 structure was validated by comparing the three-dimensional model with PROCHECK and PROSA (https://prosa.services.came.sbg.ac.at/prosa.php/, accessed on 2 March 2025) analysis results. The model’s quality was further assessed using Verify-3D (https://saves.mbi.ucla.edu/).

### 3.4. Gene Cloning and Mutant Construction

All primer pairs used in this study are listed in Table S1. Using the AprE 2709 gene sequence (Gene ID: ABU68339.1), genomic DNA from *B. licheniformis* 2709 served as a template for PCR amplification with primers P1 and P2. The 50 μL PCR reaction system contained 1 μL template DNA, 1.5 μL of each primer (10 μM), 25 μL 2× Master Mix, and 21 μL ddH_2_O. PCR cycling conditions were as follows: initial denaturation at 95 °C for 3 min, followed by 34 cycles of denaturation at 95 °C for 30 s, annealing at 55 °C for 30 s, extension at 72 °C for 4 min, and a final extension at 72 °C for 10 min. The amplified products included the promoter sequence, signal peptide, prepeptide, and mature peptide sequence of AprE 2709, facilitating the construction of the recombinant expression vector pBE2R/2709. The recombinant plasmid containing the AprE 2709 coding gene (pBE2R/2709) was used as a template for site-directed mutagenesis via the whole-plasmid PCR method. The PCR products were treated with 1 μL of DpnI for 1 h to digest methylated or hemimethylated parental templates and then transformed into *Escherichia coli* DH5α competent cells. The cells were plated on Luria–Bertani (LB) agar plates (containing 1 g of peptone, 0.5 g of yeast extract, 1 g of NaCl, and 2 g of agar powder per 100 mL of water) with 100 μg/mL ampicillin to select positive clones. Positive clones were confirmed by DNA sequencing to verify the presence of the correct mutant plasmid.

### 3.5. Heterologous Expression of AprE 2709 Wild-Type and Mutants

The recombinant plasmids were transformed into *B. subtilis* WB600 competent cells via chemical transformation [44]. *B. subtilis* WB600 containing pBE2R/2709 or its mutants were cultured overnight in 100 mL of LB medium (10 g/L peptone, 10 g/L NaCl, and 5 g/L yeast extract) with kanamycin (50 μg/mL), shaken at 37 °C and 200 rpm. The culture was then inoculated into 300 mL of fermentation medium (1 g/L dextrin, 2 g/L soluble starch, 1 g/L yeast extract, 0.5 g/L sodium chloride, 4 g/L peanut cake powder, and 0.1 g/L amylase) in a 3 L container and fermented under the same conditions for approximately 84 h at 37 °C.

### 3.6. Enzyme Activity

Alkaline protease activity was determined with the Folin–phenol method (GB/T 23527-2009) [45,46]. Briefly, 500 µL of enzyme extract and 500 µL of casein solution (10 g/L) were mixed and incubated at 40 °C for 10 min. The reaction was then stopped by adding 1 mL of 0.4 mol/L trichloroacetic acid, followed by centrifugation at 13,000× *g* for 1 min. The supernatant (1 mL) was combined with 5 mL of 0.4 mol/L Na_2_CO_3_ and 1 mL of Folin–phenol reagent and incubated at 40 °C for 20 min. Protease activity was quantified by measuring absorbance at 680 nm using a spectrophotometer. One unit of enzyme activity was defined as the amount of enzyme required to produce 1 µg of tyrosine per minute.

### 3.7. Enzymatic Properties of AprE 2709 Wild-Type and Mutants

The enzyme activity was assessed at temperatures ranging from 30 °C to 70 °C for the establishment of the optimal reaction temperature. The enzyme was incubated in a water bath at 60 °C for 60 min, after which residual activity was measured to assess thermal stability. Half-life was calculated by the exponential fitting of the data points. A series of buffer systems with a pH gradient and a concentration of 0.2 M were prepared: MES (pH 6.0), Na_2_HPO_4_–NaH_2_PO_4_ (pH 6.5–7.0), Tris-HCl (pH 8.0–9.0), and CAPS–NaOH (pH 10.0–12.0). Enzyme activity was measured in buffers with pH values ranging from 6.0 to 12.0 to determine the optimum pH for the reaction. All experiments were performed in duplicate under standard assay conditions.

### 3.8. Enzymatic Kinetic Assays

The kinetic parameters of AprE 2709 wild-type (WT) and its mutants were assessed by measuring enzyme activity at 40 °C in 50 mM Tris-HCl and 100 mM NaCl buffers (pH 8.0). Enzyme activity was measured over a 10 min period using casein concentrations ranging from 0.5 mM to 4 mM. The kinetic parameters (K_m_ and V_max_) were determined by fitting the enzyme activity data to the Michaelis–Menten equation using OriginPro (Learning Edition). Each experiment was performed independently and replicated three times to ensure the reliability and reproducibility of the results.

### 3.9. Molecular Dynamics Simulation

Molecular dynamics simulations were performed using GROMACS (Version 22.06) biomolecular simulation software with the Amber14sb all-atom force field [47]. AprE 2709 (WT) and M14 mutant forms of AprE 2709 underwent 300 ns of simulation. Each protease was solvated in a cubic box using the OPC3 three-point water model, maintaining a minimum distance of 1.0 nm between the solute and the box walls [48,49]. Approximately 10,500 water molecules were used to solvate AprE 2709 (WT) and M14 mutant, with three chloride ions added to neutralize the system’s overall positive charge. Energy minimization of the solvated structure was performed using the steepest descent method for 50,000 steps under constant conditions (without temperature coupling) until the maximum force reached below 100.0 kJ/mol/nm, optimizing the structure and eliminating steric clashes [50]. The LINCS algorithm was applied to constrain all hydrogen-heavy atom bonds [51,52]. The equilibration of the simulation system was achieved in two phases. The first phase was conducted under NVT ensemble conditions (constant number of particles, volume, and temperature) for 100 ps, allowing the system to reach temperature equilibrium at 298.15 K. The second phase, under NPT ensemble conditions (constant number of particles, pressure, and temperature), lasted 2000 ps, during which the system attained pressure equilibrium at 1 bar. Conformational analyses of the protease clusters were performed using GROMACS analysis tools and other analysis scripts, employing methods such as root-mean-square deviation (RMSD), root-mean-square fluctuation (RMSF), radius of gyration (Rg), Solvent-Accessible Surface Area (SASA), and B-factor.

### 3.10. Statistical Analysis

The exponential decay model was selected for fitting using OriginPro (Version 2021) software. The average data of residual enzyme activity and error values, measured every ten minutes at 60 °C during the thermal stability test, were input into the software. The software then applied nonlinear least squares and other algorithms to compute the best-fit curve. The half-life value was accurately determined using the equation t_1/2_ = ln2/k (where k represents the half-life constant).

## 4. Conclusions

This study successfully employed molecular modeling techniques, including AlphaFold 3 and SWISS-MODEL, to predict the three-dimensional structure of the alkaline protease AprE 2709 from *Bacillus licheniformis*. Molecular dynamics simulations revealed significant fluctuations in asparagine residues, which are susceptible to deamidation in aqueous environments. Based on this observation, site-directed mutagenesis was performed at positions 61, 160, and 211, generating a series of AprE 2709 mutants. Among these, the triple mutant M14 (AprE 2709 N61G/N160G/N211G) exhibited remarkable thermal stability and catalytic efficiency. Specifically, M14 exhibited a 2.89-fold increase in half-life at 60 °C and a 1.56-fold improvement in catalytic efficiency compared to the wild-type enzyme. Structural analysis suggested that reducing steric hindrance at the mutated sites contributed significantly to the enhanced stability. Furthermore, molecular dynamics simulations indicated that reinforced electrostatic interactions and hydrophobic effects played a crucial role in improving stability. These results provide a solid framework for the rational design of proteases with improved thermal stability and catalytic efficiency, offering valuable insights for future enzyme engineering endeavors.

## Figures and Tables

**Table 1:** RMSF scores for the 19 asparagine loci in AprE 2709 (WT).

Asparagine Sites (19 in Total)	RMSF Values (nm)
76	0.2127
160	0.1723
182	0.1631
184	0.107
96	0.105
43	0.098
154	0.0977
239	0.0898
116	0.0858
61	0.0849
211	0.0832
25	0.0753
162	0.0703
217	0.0592
140	0.0584
57	0.0579
247	0.0475
268	0.046
122	0.0443

**Table 2:** T_1/2_ value of the AprE 2709 (WT) and mutants.

Enzymes	Mutated Sites	T_1/2_ ^a^ (min)
WT	-	19
M1	N61S	29
M2	N160S	39
M3	N211S	54
M4 (M1 + M2)	N61S/N160S	30
M5 (M2 + M3)	N160S/N211S	40
M6 (M1 + M3)	N61S/N211S	41
M7 (M1 + M2 + M3)	N61S/N160S/N211S	62
M8	N61G	23
M9	N160G	15
M10	N211G	14
M11 (M8 + M9)	N61G/N160G	35
M12 (M9 + M10)	N160G/N211G	18
M13 (M8 + M10)	N61G/N211G	28
M14 (M8 + M9 + M10)	N61G/N160G/N211G	74

^a^ Half-life of enzymes after incubation at 60 °C.

**Table 3:** Enzymatic properties of AprE 2709 (WT) and mutants.

Types	K_m_ (mM)	k_cat_ (min^−1^)	k_cat_/K_m_ (mM^−1^·min^−1^)	Optimal pH	Optimum Temperature
WT	0.57	9.42	16.29	10	50 °C
M1	0.15	4.82	32.11	11	60 °C
M2	0.65	5.25	7.99	11	60 °C
M3	0.06	5.42	82.19	11	60 °C
M4	1.09	5.52	5.06	11	60 °C
M5	0.05	6.97	148.32	11	60 °C
M6	0.04	4.65	116.41	11	60 °C
M7	0.50	5.19	10.31	11	60 °C
M8	0.55	7.86	14.22	11	60 °C
M9	0.28	6.40	22.28	11	60 °C
M10	0.21	7.37	33.75	11	60 °C
M11	0.77	6.12	7.89	11	60 °C
M12	0.16	5.92	35.15	11	60 °C
M13	0.28	6.46	22.34	11	60 °C
M14	0.25	6.53	25.42	11	60 °C

## Data Availability

Data are contained within the article.

## Supplementary Materials

The following supporting information can be downloaded at https://www.mdpi.com/article/10.3390/molecules30051160/s1. Figure S1: Prediction of secondary and tertiary structure of AprE 2709 (WT). Green regions represented folded structures; orange regions depicted helical structures. Figure S2: Ramachandran plot of AprE by Procheck. Here, 90.8% of the residues were in the most favorable region, 8.8% were in the additional allowable region, and 0.4% were in a large number of allowable regions. Figure S3: Structure evaluation diagram of AprE 2709 (WT) using VERIFY-3D. Figure S4: Structure evaluation diagram of AprE 2709 (WT) using PROSA. Figure S5: Kinetic profiles of AprE 2709 (WT) and S mutants. Figure S6: Kinetic profiles of AprE 2709 (WT) and G mutants. Table S1: Primer sequences used for the construction in this article.

## References

[1] Mokashe N., Chaudhari B., Patil U. (2018). Operative utility of salt–stable proteases of halophilic and halotolerant bacteria in the biotechnology sector. Int. J. Biol. Macromol..

[2] Rozanov A.S., Shekhovtsov S.V., Bogacheva N.V., Pershina E.G., Ryapolova A.V., Bytyak D.S., Peltek S.E. (2021). Production of subtilisin proteases in bacteria and yeast. Vavilov J. Genet. Breed..

[3] Hammami A., Hamdi M., Abdelhedi O., Jridi M., Nasri M., Bayoudh A. (2017). Surfactant and oxidant stable alkaline proteases from Bacillus invictae: Characterization and potential applications in chitin extraction and as a detergent additive. Int. J. Biol. Macromol..

[4] Matkawala F., Nighojkar S., Kumar A., Nighojkar A. (2021). Microbial alkaline serine proteases: Production, properties and applications. World J. Microbiol. Biotechnol..

[5] Zhou C., Yang G., Zhang L., Zhang H., Zhou H., Lu F. (2021). Construction of an alkaline protease overproducer strain based on *Bacillus licheniformis* 2709 using an integrative approach. Int. J. Biol. Macromol..

[6] Zhou C.X., Zhang H.T., Fang H.L., Sun Y.Q., Zhou H.Y., Yang G.C., Lu F.P. (2021). Transcriptome based functional identification and application of regulator AbrB on alkaline protease synthesis in 2709. Int. J. Biol. Macromol..

[7] Zhou C., Kong Y., Zhang N., Qin W., Li Y., Zhang H., Yang G., Lu F. (2024). Regulator DegU can remarkably influence alkaline protease AprE biosynthesis in *Bacillus licheniformis* 2709. Int. J. Biol. Macromol..

[8] Zhou C., Liu H., Yuan F., Chai H., Wang H., Liu F., Li Y., Zhang H., Lu F. (2019). Development and application of a CRISPR/Cas9 system for *Bacillus licheniformis* genome editing. Int. J. Biol. Macromol..

[9] Zhou C., Zhou H., Li D., Zhang H., Wang H., Lu F. (2020). Optimized expression & enhanced production of alkaline protease by genetically modified *Bacillus licheniformis* 2709. Microb. Cell Factories.

[10] Zhou C., Yang G., Meng P., Qin W., Li Y., Lin Z., Hui W., Zhang H., Lu F. (2024). Identification and engineering of the aprE regulatory region and relevant regulatory proteins in *Bacillus licheniformis* 2709. Microb. Cell Factories.

[11] Ashraf N.M., Krishnagopal A., Hussain A., Kastner D., Sayed A.M.M., Mok Y.K., Swaminathan K., Zeeshan N. (2019). Engineering of serine protease for improved thermostability and catalytic activity using rational design. Int. J. Biol. Macromol..

[12] Zhu F., Li G., Wei P., Song C., Xu Q., Ma M., Ma J., Song P., Zhang S. (2022). Rational engineering of a metalloprotease to enhance thermostability and activity. Enzym. Microb. Technol..

[13] Peng Z., Miao Z., Ji X., Zhang G., Zhang J. (2022). Engineering flexible loops to enhance thermal stability of keratinase for efficient keratin degradation. Sci. Total Environ..

[14] Liu B., Zhang J., Fang Z., Gu L., Liao X., Du G., Chen J. (2013). Enhanced thermostability of keratinase by computational design and empirical mutation. J. Ind. Microbiol. Biotechnol..

[15] Kato K., Nakayoshi T., Kitamura Y., Kurimoto E., Oda A., Ishikawa Y. (2023). Identification of the Most Impactful Asparagine Residues for γS- Crystallin Aggregation by Deamidation. Biochemistry.

[16] Shi Y., Rhodes N.R., Abdolvahabi A., Kohn T., Cook N.P., Marti A.A., Shaw B.F. (2013). Deamidation of Asparagine to Aspartate Destabilizes Cu, Zn Superoxide Dismutase, Accelerates Fibrillization, and Mirrors ALS-Linked Mutations. J. Am. Chem. Soc..

[17] Hains P.G., Truscott R.J. (2010). Age-dependent deamidation of lifelong proteins in the human lens. Investig. Ophthalmol. Vis. Sci..

[18] Pace A.L., Wong R.L., Zhang Y.T., Kao Y.H., Wang Y.J. (2013). Asparagine deamidation dependence on buffer type, pH, and temperature. J. Pharm. Sci..

[19] Rahimzadeh M., Khajeh K., Mirshahi M., Khayatian M., Schwarzenbacher R. (2012). Probing the role of asparagine mutation in thermostability of Bacillus KR-8104 α-amylase. Int. J. Biol. Macromol..

[20] Declerck N., Machius M., Wiegand G., Huber R., Gaillardin C. (2000). Probing structural determinants specifying high thermostability in *Bacillus licheniformis* α-amylase. J. Mol. Biol..

[21] Bhanuramanand K., Ahmad S., Rao N.M. (2014). Engineering deamidation-susceptible asparagines leads to improved stability to thermal cycling in a lipase. Biochem. Biophys. Res. Commun..

[22] Bandi S., Singh S.M., Shah D.D., Upadhyay V., Mallela K.M.G. (2019). 2D NMR Analysis of the Effect of Asparagine Deamidation Versus Methionine Oxidation on the Structure, Stability, Aggregation, and Function of a Therapeutic Protein. Mol. Pharmacist..

[23] Yuan S.S., Yan R.X., Lin B.Y., Li R.K., Ye X.Y. (2023). Improving thermostability of *Bacillus amyloliquefaciens* alpha-amylase by multipoint mutations. Biochem. Biophys. Res. Commun..

[24] Zhao J.-F., Wang Z., Gao F.-L., Lin J.-P., Yang L.-R., Wu M.-B. (2018). Enhancing the thermostability of Rhizopus oryzae lipase by combined mutation of hot-spots and engineering a disulfide bond. RSC Adv..

[25] Aghaeepoor M., Akbarzadeh A., Mirzaie S., Hadian A., Aval S.J., Dehnavi E. (2018). Selective reduction in glutaminase activity of L-Asparaginase by asparagine 248 to serine mutation: A combined computational and experimental effort in blood cancer treatment. Int. J. Biol. Macromol..

[26] Kamble A., Singh R., Singh H. (2025). Structural and Functional Characterization of Eubacterium proteus Phytase: A Comprehensive In-Silico Study. Mol. Biotechnol..

[27] Min K., Kim H., Park H.J., Lee S., Jung Y.J., Yoon J.H., Lee J.S., Park K., Yoo Y.J., Joo J.C. (2021). Improving the catalytic performance of xylanase from *Bacillus circulans* through structure-based rational design. Bioresour. Technol..

[28] Amatto I.V.d.S., da Rosa-Garzon N.G., de Oliveira Simões F.A., Santiago F., da Silva Leite N.P., Martins J.R., Cabral H. (2022). Enzyme engineering and its industrial applications. Biotechnol. Appl. Biochem..

[29] Su H.H., Peng F., Xu P., Wu X.L., Zong M.H., Yang J.G., Lou W.Y. (2019). Enhancing the thermostability and activity of uronate dehydrogenase from LBA4404 by semi-rational engineering. Bioresour. Bioprocess..

[30] Lv K., Li X., Chen K., Wu B., He B., Schenk G. (2024). Improving the Catalytic Efficiency of a GH5 Processive Endoglucanase by a Combinatorial Strategy Using Consensus Mutagenesis and Loop Engineering. ACS Catal..

[31] Lee C.Y., Yu K.O., Kim S.W., Han S.O. (2010). Enhancement of the thermostability and activity of mesophilic EngD by DNA recombination with CelE. J. Biosci. Bioeng..

[32] Zhao G., Wang J.R., Tang Q.Y., Lan D.M., Wang Y.H. (2018). Improving the Catalytic Activity and Thermostability of MAS1 Lipase by Alanine Substitution. Mol. Biotechnol..

[33] Wu X., Deng F., Chen Y., Xu M., Ma F., Shi L. (2024). Electrostatic and hydrophobic interaction cooperative nanochaperone regulates protein folding. Aggregate.

[34] Baldwin R.L., Rose G.D. (2016). How the hydrophobic factor drives protein folding. Proc. Natl. Acad. Sci. USA.

[35] Yi Z.L., Pei X.Q., Wu Z.L. (2011). Introduction of glycine and proline residues onto protein surface increases the thermostability of endoglucanase CelA from *Clostridium thermocellum*. Bioresour. Technol..

[36] Li Y., Wei L., Zhu Z., Li S., Wang J.W., Xin Q., Wang H., Lu F., Qin H.M. (2017). Rational design to change product specificities and thermostability of cyclodextrin glycosyltransferase from *Paenibacillus* sp. RSC Adv..

[37] Lee J.M., Moon S.Y., Kim Y.R., Kim K.W., Lee S.J., Kong I.S. (2017). Improvement of thermostability and halo stability of β-1,3-1,4-glucanase by substituting hydrophobic residue for Lys. Int. J. Biol. Macromol..

[38] Gaines J.C., Clark A.H., Regan L., O’Hern C.S. (2017). Packing in protein cores. J. Phys. Condens. Matter.

[39] Nguyen C., Young J.T., Slade G.G., Oliveira R.J., McCully M.E. (2019). A Dynamic Hydrophobic Core and Surface Salt Bridges Thermostabilize a Designed Three-Helix Bundle. Biophys. J..

[40] Taylor T.J., Vaisman I.I. (2010). Discrimination of thermophilic & mesophilic proteins. BMC Struct. Biol..

[41] Cui X., Yuan X., Li S.Y., Hu X.L., Zhao J., Zhang G.M. (2022). Simultaneously improving the specific activity and thermostability of α-amylase BLA by rational design. Bioprocess Biosyst. Eng..

[42] Waterhouse A., Bertoni M., Bienert S., Studer G., Tauriello G., Gumienny R., Heer F.T., de Beer T.A.P., Rempfer C., Bordoli L. (2018). SWISS-MODEL: Homology modelling of protein structures and complexes. Nucleic Acids Res..

[43] Aderinwale T., Bharadwaj V., Christoffer C., Terashi G., Zhang Z., Jahandideh R., Kagaya Y., Kihara D. (2022). Real-time structure search and structure classification for AlphaFold protein models. Commun. Biol..

[44] Tang X.M., Shen W., Lakay F.M., Shao W.L., Wang Z.X., Prior B.A., Zhuge J. (2004). Cloning and over-expression of an alkaline protease from *Bacillus licheniformis*. Biotechnol. Lett..

[45] Hiraga K., Nishikata Y., Namwong S., Tanasupawat S., Takada K., Oda K. (2005). Purification and characterization of serine proteinase from a *Halophilic bacterium*, sp. RF2-5. Biosci. Biotechnol. Biochem..

[46] (2009). Proteinase Preparations.

[47] Siu S.W.I., Pluhackova K., Böckmann R.A. (2012). Optimization of the OPLS–AA Force Field for Long Hydrocarbons. J. Chem. Theory Comput..

[48] Fyta M., Netz R.R. (2012). Ionic force field optimization based on single-ion and ion-pair solvation properties: Going beyond st and ard mixing rules. Chin. J. Chem. Phys..

[49] Kusalik P.G., Svishchev I.M. (1994). The Spatial Structure in Liquid Water. Science.

[50] Hess B. (2008). P–LINCS: A parallel linear constraint solver for molecular simulation. J. Chem. Theory Comput..

[51] Hess B., Bekker H., Berendsen H.J.C., Fraaije J.G.E.M. (1997). LINCS: A linear constraint solver for molecular simulations. J. Comput. Chem..

[52] Ullah S., Khan S.U., Khan A., Junaid M., Rafiq H., Htar T.T., Zhao Y.X., Shah S.A.A., Wadood A. (2022). Prospect of Anterior Gradient 2 homodimer inhibition via repurposing FDA–approved drugs using structure–based virtual screening. Mol. Divers..

