# Supplementary material for: Enhancing the Thermostability of Bacillus licheniformis Alkaline Protease 2709 by Computation-Based Rational Design
Source: Molecules. 2025 Mar 4;30(5):1160. doi: 10.3390/molecules30051160 (PMC11901772; doi:10.3390/molecules30051160)
Supplement: Supplementary file 1 [file molecules-30-01160-s001.zip › molecules-3455826-supplementary.pdf]

Supporting Information

# Enhancing the Thermostability of *Bacillus licheniformis* Alkaline Protease 2709 by Computation-Based Rational Design

**Yuan Yuan [1], Guowei Zhao [1], Jing Lu [2], Lei Wang [3], Yawei Shi [2,4,*] and Jian Zhang [1,4,*]**

[1] College of Chemistry and Chemical Engineering, Shanxi University, Taiyuan 030006, China; 202112910002@email.sxu.edu.cn (Y.Y.); 202122911025@email.sxu.edu.cn (G.Z.)
[2] College of Life Sciences, Shanxi University, Taiyuan 030006, China; jinglu@sxu.edu.cn
[3] Key Laboratory of Chemical Biology and Molecular Engineering, Ministry of Education, Institute of Biotechnology, Shanxi University, Taiyuan 030006, China; wanglei1007@sxu.edu.cn
[4] Shanxi Province Detergent Alkaline Protease Industrialization Key Technology and Application Engineering Research Center, Taiyuan 030006, China
* Correspondence: yaweishi@sxu.edu.cn (Y.S.); zhangjian@sxu.edu.cn ; Tel.: +86-138-0345-1349 (Y.S.); +86-138-3411-0276 (J.Z.)

Figure.S1 Prediction of secondary and tertiary structure of AprE 2709 (WT). Green regions represented folded structures; orange regions depicted helical structures.

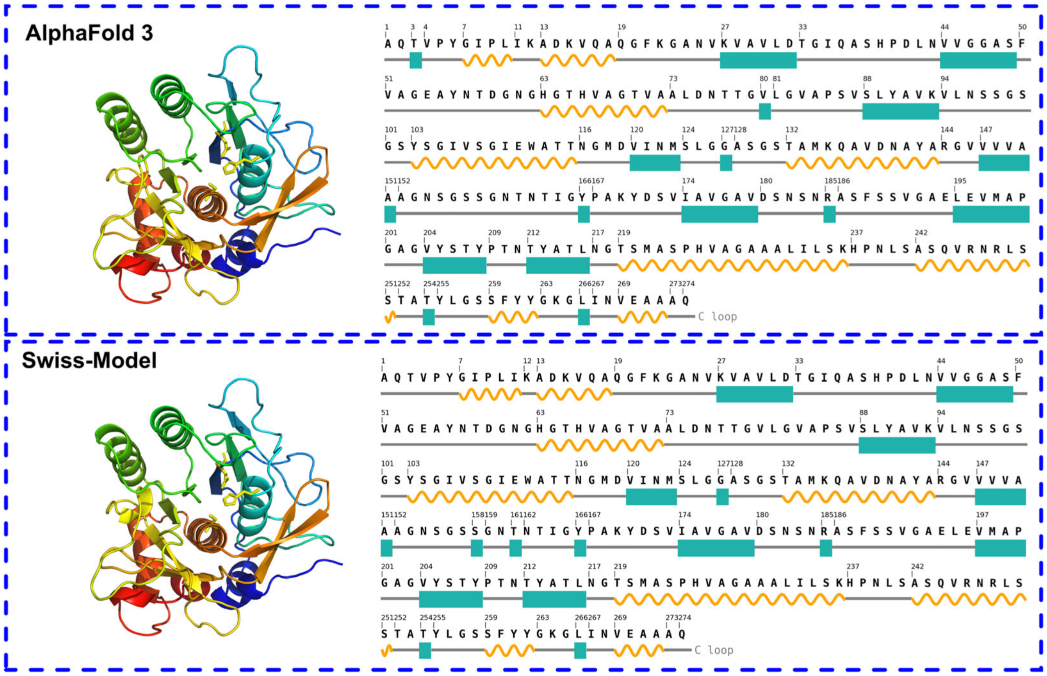

Figure S2 Ramachandran plot of AprE by Procheck. 90.8% of the residues were in the most favorable region, 8.8% were in the additional allowable region, and 0.4% were in a large number of allowable regions.

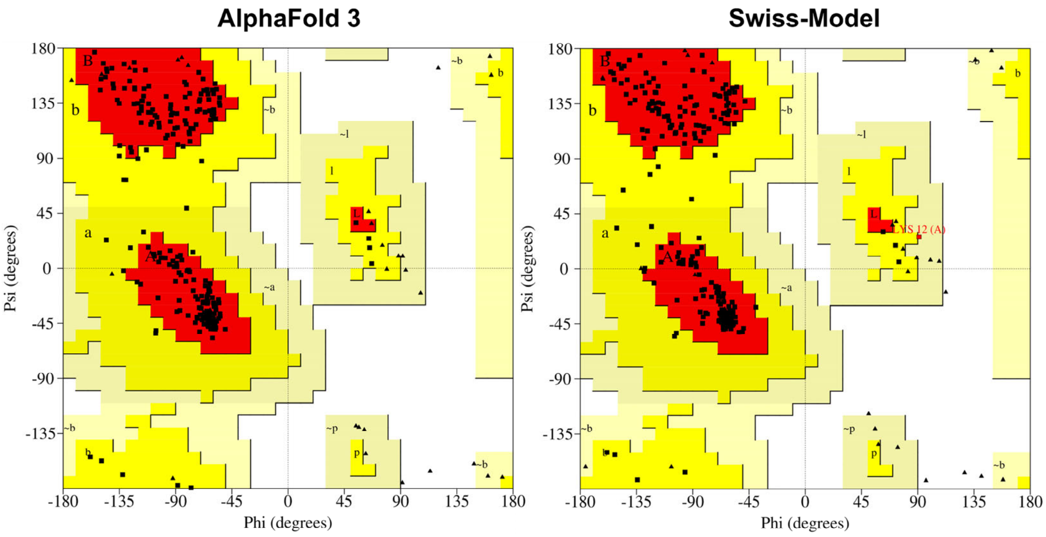


AlphaFold 3 — Plot statistics

| | | |
|---|---|---|
| Residues in most favoured regions [A,B,L] | 203 | 89.0% |
| Residues in additional allowed regions [a,b,l,p] | 25 | 11.0% |
| Residues in generously allowed regions [~a,~b,~l,~p] | 0 | 0.0% |
| Residues in disallowed regions | 0 | 0.0% |
| | ---- | ------ |
| Number of non-glycine and non-proline residues | 228 | 100.0% |
| Number of end-residues (excl. Gly and Pro) | 2 | |
| Number of glycine residues (shown as triangles) | 35 | |
| Number of proline residues | 9 | |
| | ---- | |
| Total number of residues | 274 | |

Swiss-Model — Plot statistics

| | | |
|---|---|---|
| Residues in most favoured regions [A,B,L] | 207 | 90.8% |
| Residues in additional allowed regions [a,b,l,p] | 20 | 8.8% |
| Residues in generously allowed regions [~a,~b,~l,~p] | 1 | 0.4% |
| Residues in disallowed regions | 0 | 0.0% |
| | ---- | ------ |
| Number of non-glycine and non-proline residues | 228 | 100.0% |
| Number of end-residues (excl. Gly and Pro) | 5 | |
| Number of glycine residues (shown as triangles) | 35 | |
| Number of proline residues | 9 | |
| | ---- | |
| Total number of residues | 277 | |

Figure S3 Structure evaluation diagram of AprE 2709 (WT) using VERIFY-3D.

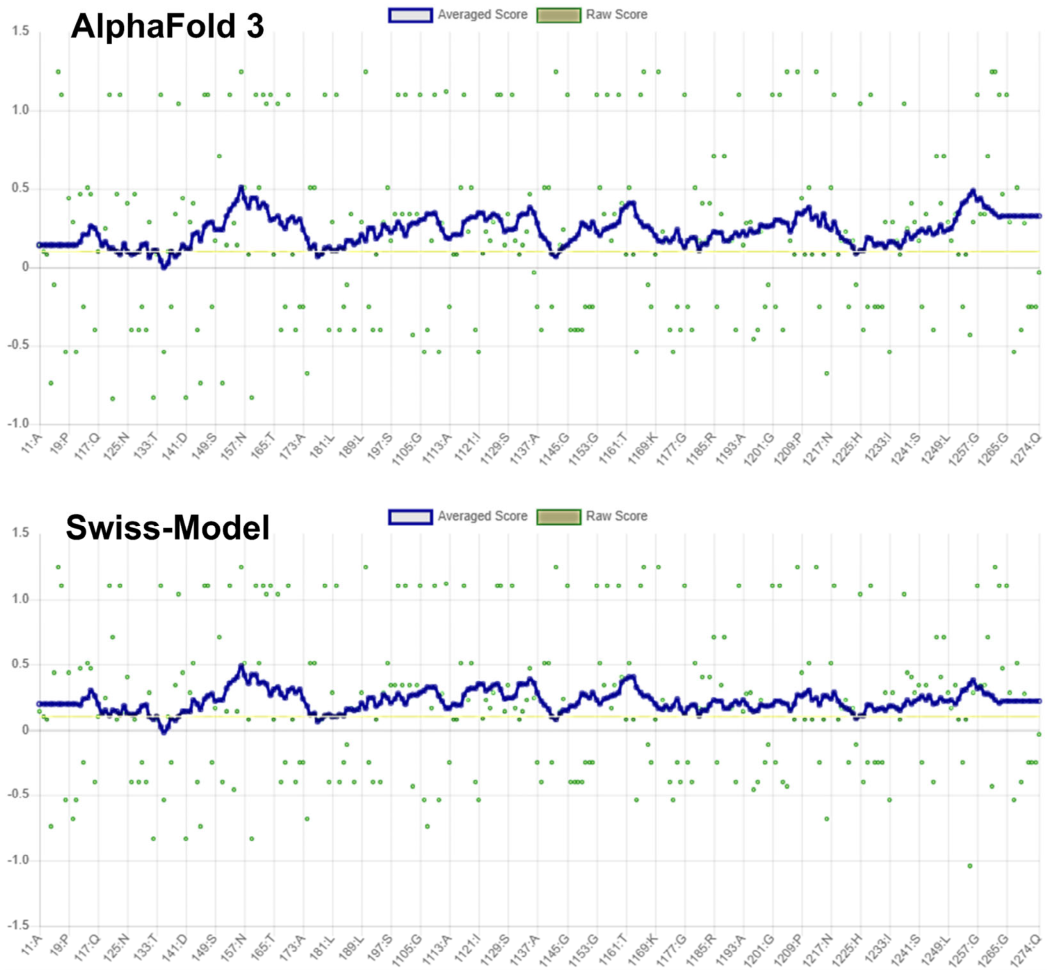

Figure S4 Structure evaluation diagram of AprE 2709 (WT) using PROSA.

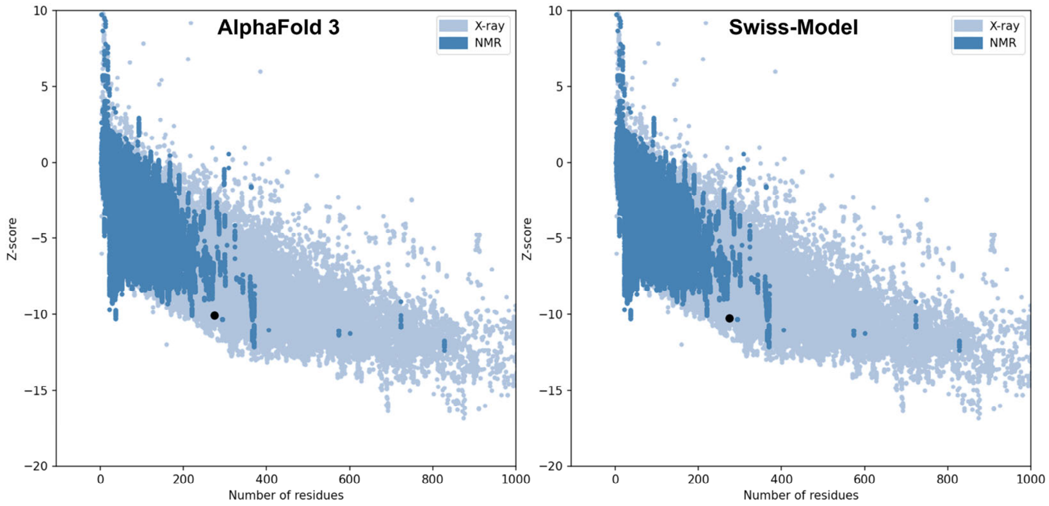

Figure S5 Kinetic profiles of AprE 2709 (WT) and S mutants.

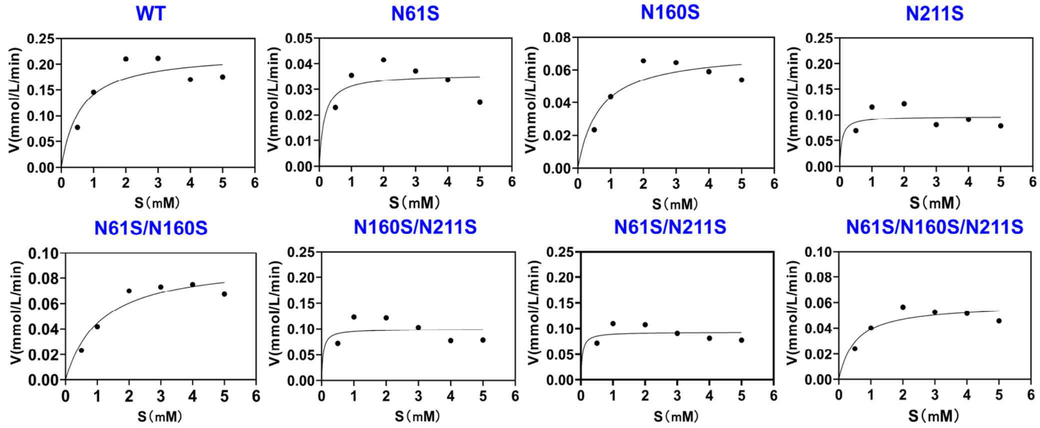

Figure S6 Kinetic profiles of AprE 2709 (WT) and G mutants.

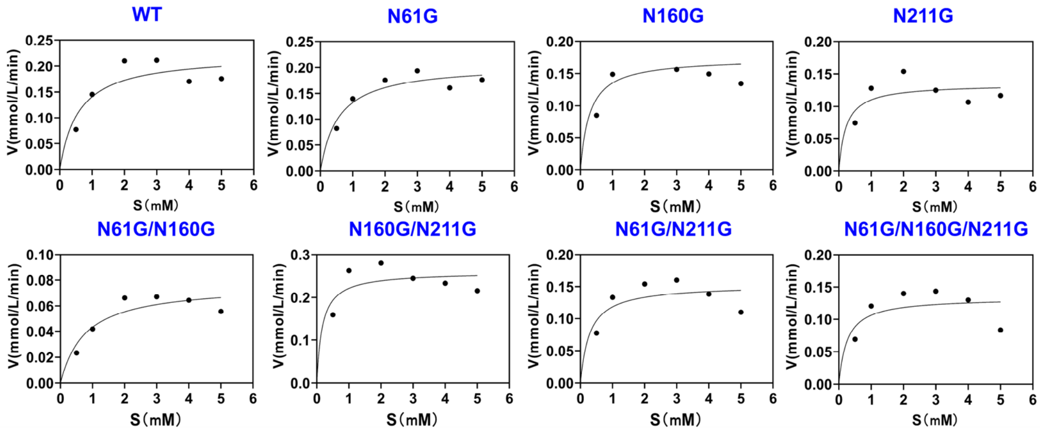

Table. S1 primer sequences used for the construction in this article

| Primer name | Nucleotide sequence (5'-3') |
|---|---|
| P1 | TGTACGTTCCTTAAGGAATTCACAAGGCCGCAACCTCCT |
| P2 | GCAGGTCGACTCTAGAGGATCCTTATTGAGCGGCAGCTTC |
| N61G-F | TAACACCGACGGCGGCGGACACGGCACAC |
| N61G-R | CCGCCGTCGGTGTTATAAGCTTCGCC |
| N61S-F | TAACACCGACGGCAGCGGACACGGCACAC |
| N61S-R | CTGCCGTCGGTGTTATAAGCTTCGCC |
| N160G-F | GCGGATCTTCAGGAGGCACGAATACAATT |
| N160G-R | CCTCCTGAAGATCCGCTGTTCCCTGC |
| N160S-F | GCGGATCTTCAGGAAGCACGAATACAATT |
| N160S-R | CTTCCTGAAGATCCGCTGTTCCCTGC |
| N211G-F | GCACTTACCCAACGGGCACTTATGCAACAT |
| N211G-R | CCCGTTGGGTAAGTGCTGTATACGCCT |
| N211S-F | GCACTTACCCAACGAGCACTTATGCAACA |
| N211S-R | CTCGTTGGGTAAGTGCTGTATACGCCT |
